# A Multimodal IoT-Based Locomotion Classification System Using Features Engineering and Recursive Neural Network

**DOI:** 10.3390/s23104716

**Published:** 2023-05-12

**Authors:** Madiha Javeed, Naif Al Mudawi, Bayan Ibrahimm Alabduallah, Ahmad Jalal, Wooseong Kim

**Affiliations:** 1 Department of Computer Science, Air University, Islamabad 44000, Pakistan; 2 Department of Computer Science, College of Computer Science and Information System, Najran University, Najran 55461, Saudi Arabia; 3 Department of Information Systems, College of Computer and Information Sciences, Princess Nourah Bint Abdulrahman University, P.O. Box 84428, Riyadh 11671, Saudi Arabia; 4 Department of Computer Engineering, Gachon University, Seongnam 13120, Republic of Korea

**Keywords:** activities of daily living classification, ambient sensor, inertial filter, multimodal locomotion, locomotion prediction, visual sensors

## Abstract

Locomotion prediction for human welfare has gained tremendous interest in the past few years. Multimodal locomotion prediction is composed of small activities of daily living and an efficient approach to providing support for healthcare, but the complexities of motion signals along with video processing make it challenging for researchers in terms of achieving a good accuracy rate. The multimodal internet of things (IoT)-based locomotion classification has helped in solving these challenges. In this paper, we proposed a novel multimodal IoT-based locomotion classification technique using three benchmarked datasets. These datasets contain at least three types of data, such as data from physical motion, ambient, and vision-based sensors. The raw data has been filtered through different techniques for each sensor type. Then, the ambient and physical motion-based sensor data have been windowed, and a skeleton model has been retrieved from the vision-based data. Further, the features have been extracted and optimized using state-of-the-art methodologies. Lastly, experiments performed verified that the proposed locomotion classification system is superior when compared to other conventional approaches, particularly when considering multimodal data. The novel multimodal IoT-based locomotion classification system has achieved an accuracy rate of 87.67% and 86.71% over the HWU-USP and Opportunity++ datasets, respectively. The mean accuracy rate of 87.0% is higher than the traditional methods proposed in the literature.

## 1. Introduction

From simple hand movements to complex activities of daily living (ADL), it is now possible for intelligent systems to recognize human actions by collecting the action properties from multimodal IoT-based sensors [1,2,3]. These human actions are collectively known as locomotion and can be performed in different environmental setups, such as rehabilitation centers, smart homes, and healthcare facilities [4,5,6]. The industry has advanced towards the implementation of such locomotion prediction systems as fitness trackers or fall recognition systems [7,8]. Nonetheless, such devices are based on a single sensor type and do not serve the purpose as efficiently as required [9]. Thereby, this study will fulfill the two main needs of such ADL recognition and locomotion prediction systems: enhancing the system’s accuracy and enabling the system to deal with complex motion via multimodal IoT-based locomotion classification. The applications of this system include healthcare systems, smart homes, IoT-based facilities, robotic learning, ambient assistive living, and patient monitoring.

Locomotion prediction consists of multiple locomotive abilities in humans to perform daily routine activities. Most conventional systems have used one type of sensor to classify the locomotive activities of daily living. This novel technique has used a new way of multi-sensor fusion and a skeleton model to predict human locomotion, including motion data, ambient data, and videos. This fusion of multi-sensors for locomotion prediction is referred to as the multimodal approach and can help in creating predictions that are more accurate.

Two benchmarked IoT-based datasets have been selected to verify the proposed multimodal locomotion classification system. Three types of sensor data have been utilized from these two datasets, including wearable motion, ambient, and visual sensors. At the start, we propose that the multimodal IoT-based data be filtered through different techniques that will cater to the unique requirements of noise removal in each sensor’s nature. Next, the motion and ambient filtered signals are windowed, and video frame sequences are used to extract the skeleton model. Then, the features are extracted for each sensor type separately, followed by fusing them over time series. Further, the fused features have been optimized, and locomotion via ADL is recognized. Lastly, the HWU-USP (Heriot-Watt University/University of Sao Paulo) and Opportunity++ datasets are utilized for evaluation of the proposed multimodal IoT-based locomotion classification system. Following are the key contributions of our proposed locomotion prediction system:

- Fusing the three different types of sensor data features for multimodal locomotion prediction.
- Accurate skeleton modeling for the extracted human silhouette was validated through confidence levels and skeleton point accuracies.
- Major enhancement in the accuracy of locomotion classification with improved human skeleton point confidence levels by applying a combination of different feature extraction methods and feature fusion in the proposed system methodology.

A previous systems review has been offered in Section 2, and a thorough architecture discussion about the proposed multimodal IoT-based locomotion classification model has been given in Section 3. The experiments performed have also been mentioned in Section 4, and the paper’s conclusion, along with limitations and future work discussion, has been presented in Section 5.

## 2. Literature Review

There are several studies proposed by the researchers in the literature. Some are based on either sensor-based models or vision-based methods, while others consist of multimodal systems. Section 2.1 describes sensor- or vision-based systems, and Section 2.2 gives detail about multimodal systems presented by researchers.

### 2.1. Sensor or Vision-Based Systems

Vision-based data has been used as a methodology for IoT-based locomotion classification systems. Simple inertial data has also been utilized as a monomodal system for locomotion prediction. Different investigators have offered diverse systems for locomotion prediction in the literature. In [10], a locomotion prediction system has been proposed consisting of noise removal, feature extraction, pattern retrieval, and hidden Markov model (HMM)-based classification steps. However, the locomotion detected was based on limited environmental settings and activity diversity. A study based on kinematic motion sensors has been given in Figueiredo et al. [11]. Multiple ADLs have been recognized, such as ascend stairs, descend stairs, level-ground walking, descend stairs, ascend ramp, descend ramp, etc. They have utilized feature processing, machine learning, feature reduction, and classification methods. Though the proposed study achieved good accuracy results, it did not filter the noise from raw sensor data, causing problematic outcomes. In [12], Javeed et al. described a system for locomotion prediction by introducing a calibration-based filter and a pattern identification technique. Additionally, a sliding overlapping technique was used for window extraction, and two datasets were utilized to perform experiments on the system. However, the lack of good features caused lower accuracy rates. In Wang et al. [13], a study has explained the locomotion activity recognition method for different activities. They have used radio sensor data to recognize locomotion and transportation actions. Moreover, they faced technical challenges, such as sensor unavailability and data synchronization, causing the system F1 scores to be less than 80%.

### 2.2. Multimodal Systems

Multimodal systems have opened up more challenges for locomotion prediction models. In [14], Chavarriaga et al. proposed a multimodal system based on ambient and wearable sensors. They have recognized the ADL based on modes of locomotion and gestures. Further, dynamic multimodal data fusion has enabled the system to perform better. However, the system was not able to make use of the visual sensors. In a study proposed by Ordóñez et al., complex motor activity sequences have been recognized using convolutional and long short-term memory (LSTM) recurrent neural networks [15]. Multiple layers of convolution, dense, and softmax have been used. However, due to the lack of filtration techniques applied to multimodal data, the system’s performance was low. A multimodal approach for in-home, fine-grained locomotion detection has been proposed in [16]. A two-level supervised classifier and a modified conditional random field-based supervised classifier are being utilized to recognize 19 complex IoT-based ADL. Though the system did not use any hand-crafted features, resulting in low accuracy, in [17], multiple inertial measurement units (IMU) have been used at different positions on the body to recognize human actions. A LSTM network has been produced to support the training process, and multiple ADL have been used to test the performance of the system. However, due to the lack of proper filtration and feature extraction techniques applied, the system was not able to recognize the ADL correctly.

## 3. Materials and Methods

### 3.1. System Methodology

**Figure 1:**
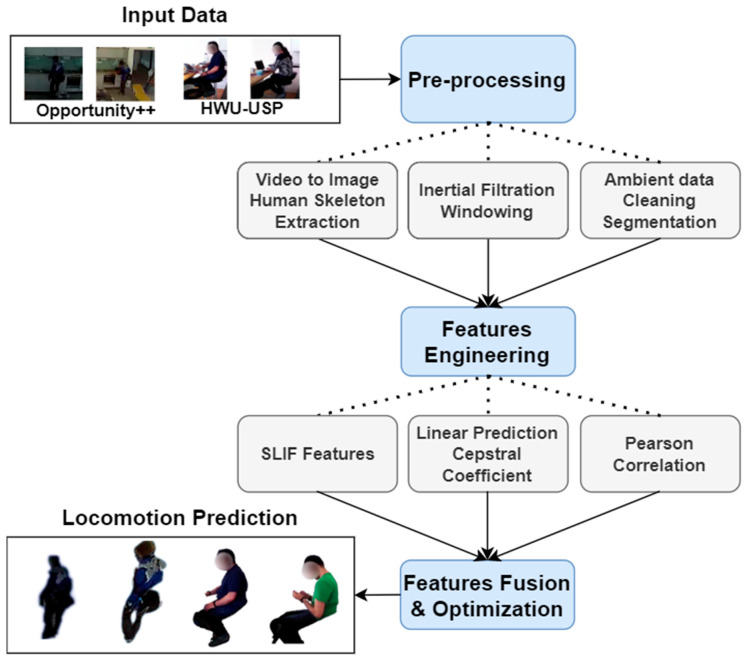
Architecture flow diagram of the proposed IoT-based multimodal locomotion prediction.

The section explains the methods used for a multimodal IoT-based locomotion classification system. The proposed locomotion prediction method first uses the multimodal raw data from two selected datasets. Next, the input data is pre-processed using three types of filters [18]. Then, the features for each sensor type are extracted using different techniques. Further, the extracted features are fused and optimized to reduce the vector dimensionality. Finally, the features are provided to the recursive neural network for locomotion classification. Figure 1 contributes to a detailed view of the architecture flow diagram proposed for this research paper.

### 3.2. IoT-Based Multimodal Data Pre-Processing

**Figure 2:**
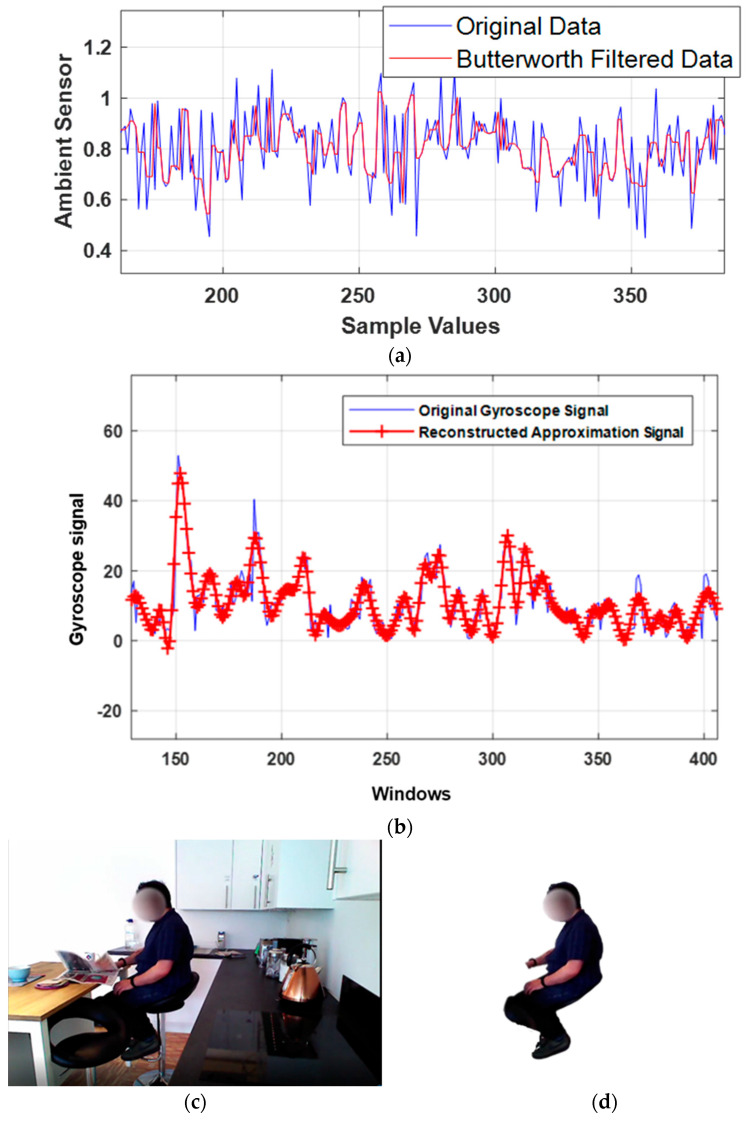
Sample signals after filters applied for (**a**) ambient sensor data, (**b**) motion sensor data, (**c**) original visual frame sequence, and (**d**) background removed frame sequence over the HWU-USP dataset.

**Figure 3:**
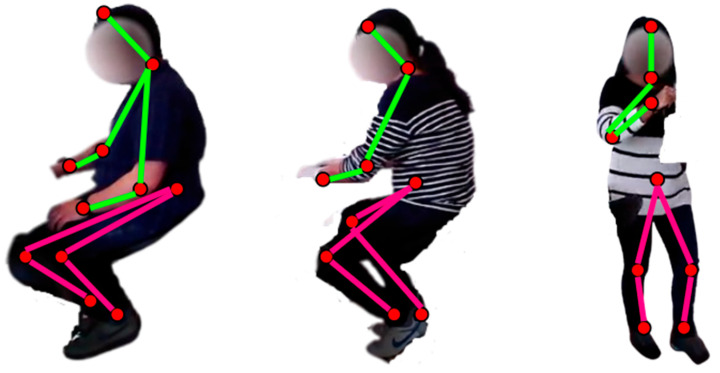
Skeleton model extracted from different sample frame sequences over the HWU-USP dataset.

First, the ambient data has been pre-processed through the Butterworth [19] filter and displayed in Figure 2a. Then, we applied a wavelet quaternion-based filter [20] over the wearable motion sensor data and demonstrated the results in Figure 2b. Both ambient and motion sensor-filtered data are windowed for 4 s [21] each to further process the signals proficiently. Conversely, the visual data has been pre-processed by subtracting the common background [22] among all subjects, as shown in Figure 2c,d. It is further utilized to extract the skeleton model [23] from the human silhouette by extracting eleven skeleton points, including the head, neck, torso, elbows, wrists, knees, and ankles. Figure 3 describes the human skeleton modelling in detail over the HWU-USP dataset.

### 3.3. Features Engineering

This section will explain three different feature extraction methods used for three types of multimodal IoT-based data. Features are extracted according to the nature of the data and the characteristics required to be mined from all the multimodal data types [24,25]. Furthermore, to improve the correctness rate of the proposed method, the selection of the most efficient feature engineering techniques, such as Pearson correlation, linear prediction cepstral coefficients, and spider local image features, is significant. The following subsections will explain the three techniques in detail.

#### 3.3.1. Pearson Correlation

**Figure 4:**
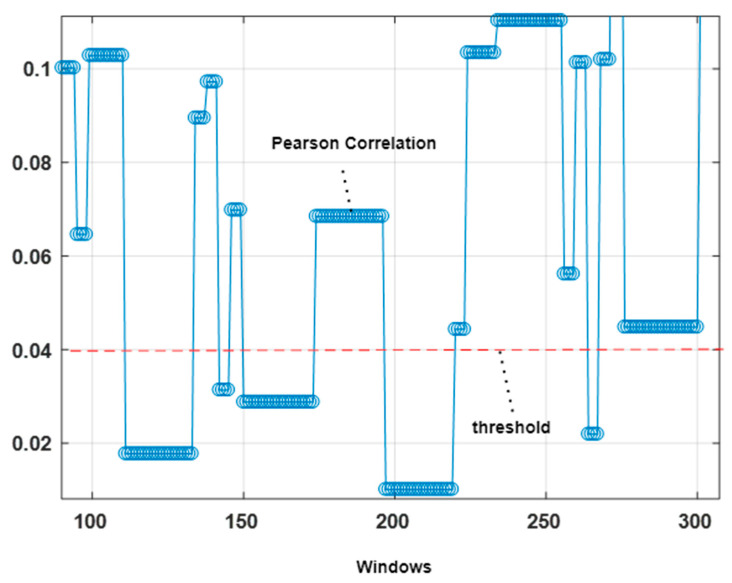
Locomotion identification using Pearson correlation.

The four-second windowed data from the ambient sensor has been used to get the actions performed features. If the average Pearson correlation [26] for a selected window is over a particular threshold of 0.04, then it is considered an action performed. Pearson correlation can be calculated as:

(1) $$PC=\frac{\sum \left(g_{x}-\bar{g}\right)\left(h_{x}-\bar{h}\right)}{\sqrt{\sum {\left(g_{x}-\bar{g}\right)}^{2}\sum {\left(h_{x}-\bar{h}\right)}^{2}}}$$

where $g_{x}$ gives the first variable value in a four second window, $h_{x}$ provides the second variable values in the window, and $\bar{g}$ and $\bar{h}$ are the mean of the first and second variable values. Figure 4 shows the Pearson correlation and a chosen threshold value with a red dashed line.

#### 3.3.2. Linear Prediction Cepstral Coefficients (LPCC)

**Figure 5:**
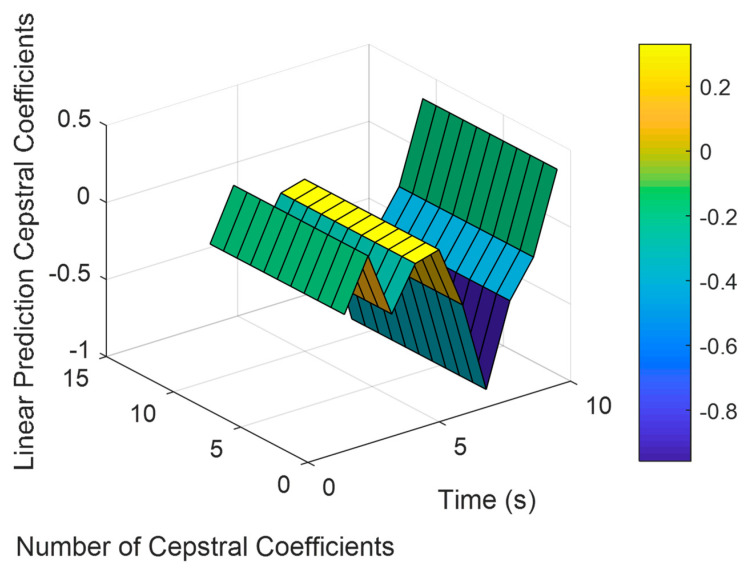
Result of LPCC applied to motion sensor-filtered and windowed data over the HWU-USP dataset.

The motion signal and its transfer function can support the extraction of LPCC. The rate of change over different bands has been declared by the cepstrum calculation [27]. Linear prediction coefficients $c_{x}$ are used to calculate the LPCC as:

(2) $$c_{x}=a_{x}+\sum_{t=1}^{x-1}\left(\frac{t}{x}\right)\;c_{t}\;a_{x-t},\;\;\;\;1\;\leq x\;\leq p,$$

(3) $$c_{x}=\sum_{t=1}^{x-1}\left(\frac{t}{x}\right)\;c_{t}\;a_{x-t},\;\;\;\;p\;\leq x\;\leq d,$$

where $a_{x}$ is the linear prediction coefficient and d presents the number of coefficients [28]. Figure 5 demonstrates the LPCC extracted for ADL, called setting the table, from the HWU-USP dataset.

#### 3.3.3. Spider Local Image Features

**Figure 6:**
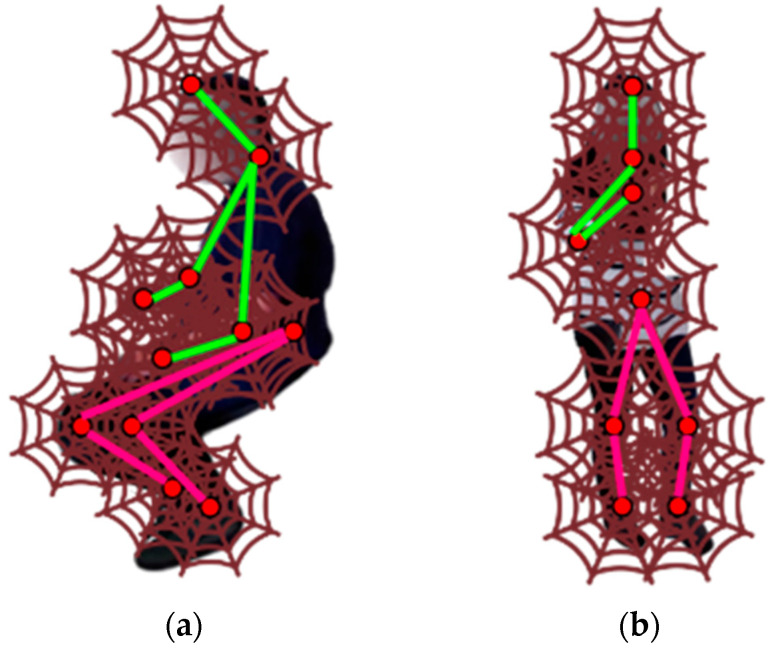
SLIF feature extraction: (**a**) reading the newspaper, (**b**) making tea over the HWU-USP dataset.

After the skeleton modeling from the human silhouette, we applied the technique called spider local image feature (SLIF). The skeleton points have been used as the web intersection points in an image [29]. The 2D coordinates are utilized to represent the x,y coordinates as:

(4) $$s_{x,z}=\left(\frac{x.cos\left(\frac{2\pi z}{Z}\right)}{X},\;\frac{x.sin\left(\frac{2\pi z}{Z}\right)}{X}\right),$$

where $\frac{x.cos\left(\frac{2\pi z}{Z}\right)}{X}$ and $\frac{x.sin\left(\frac{2\pi z}{Z}\right)}{X}$ give the horizontal and vertical coordinates. Figure 6 displays the SLIF applied over 11 skeleton points.

### 3.4. Features Fusion and Optimization

This section will explain the fusion between three types of multimodal data fusion along with the optimization technique applied to reduce the dimensionality for a large number of features.

#### 3.4.1. Features Fusion

**Figure 7:**
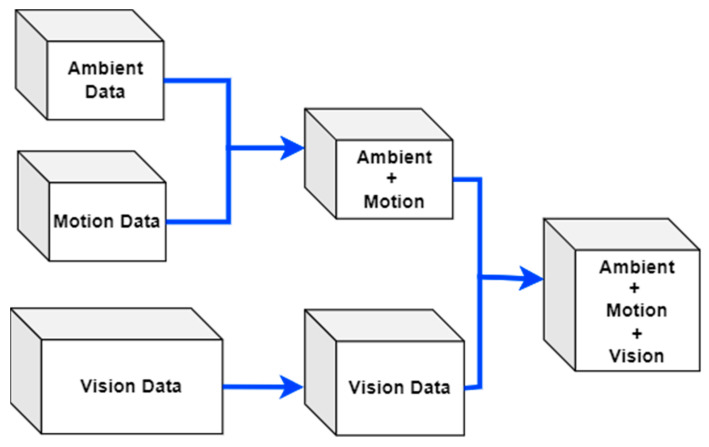
Multimodal IoT-based data feature fusion for locomotion prediction.

The featured data from ambient and motion sensors has been fused using the windows. Then, the features extracted from visual frame sequences have been fused with the ambient and motion data using skeleton points detected over the time windows in the motion data [30]. Figure 7 exhibits the feature fusion technique applied to the proposed multimodal IoT-based locomotion prediction system.

#### 3.4.2. Features Optimization

**Figure 8:**
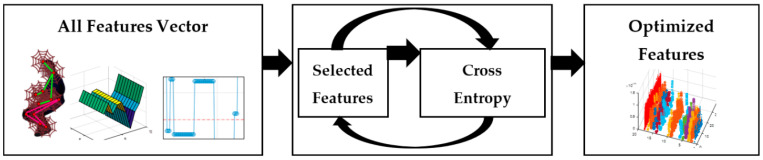
Process to obtain an optimized vector of selected features.

A cross entropy-based optimization has been applied to the large feature vector. It has selected the correlated features from the fused feature vector and reduced the vector size for deep learning-based classifier training. The mechanism is based on fuzzy logic over type-2 and a logical hierarchy procedure based additive ratio valuation [31]. Figure 8 shows the process to obtain an optimized vector for selected features.

### 3.5. Locomotion Classification via Recursive Neural Network

**Figure 9:**
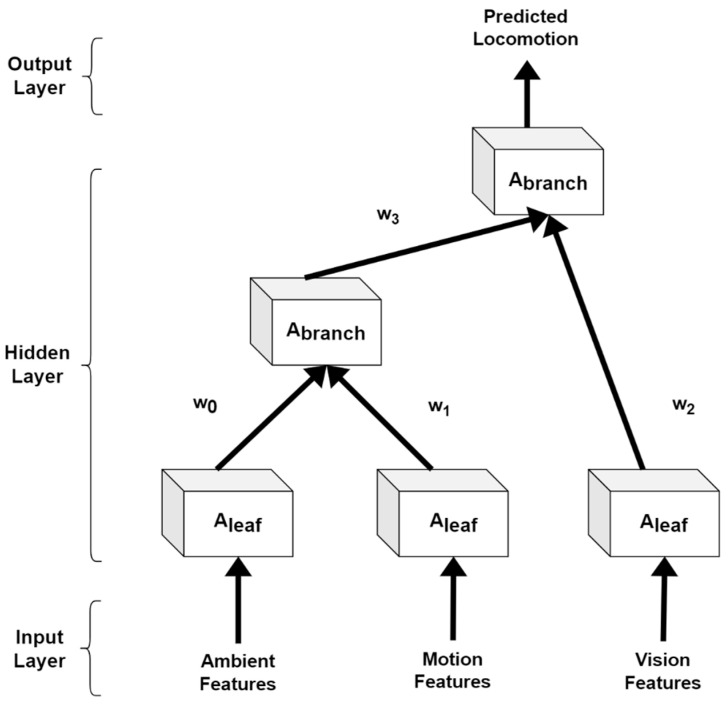
Architecture diagram for a recursive neural network.

A recursive neural network (RvNN) [32] is a deep learning-based neural network where we apply the same set of weights recursively over ambient, motion, and vision data features. We have applied this network because RvNN can be used to learn distributed, structured data. Therefore, it is ideal for our proposed multimodal IoT-based locomotion prediction system. The parent n-dimensional vector can be calculated as:

(5) $$A_{branch\left(n\right)}=\tanh\left(W\left[A_{leaf1};A_{leaf\left(n\right)}\right]\right),$$

where W represents the n×2n weight matrix and $A_{leaf\left(n\right)}$ gives the first hidden layer of RvNN. Figure 9 demonstrates the architecture used for RvNN in detail.

## 4. Experimental Setup and Evaluation

We have explained the details of the experiments performed and the validation techniques utilized in this section. Locomotion classification accuracy has been used for the performance validation of the proposed multimodal IoT-based locomotion classification system from the chosen challenging indoor datasets. The software Matlab, hardware Intel Core i7 at 2.4 GHz along with 24 GB of RAM are utilized to assess the proposed system. Two indoor activities-based datasets known as HWU-USP [33] and Opportunity++ [34] are utilized for system performance measurement. A 10-fold cross-validation technique has been used over the dataset for training purposes. Following is a description of the datasets used, their investigational outcomes, and a comparison of our proposed multimodal IoT-based locomotion classification system with conventional approaches.

### 4.1. Dataset Descriptions

#### 4.1.1. HWU-USP Dataset

**Figure 10:**
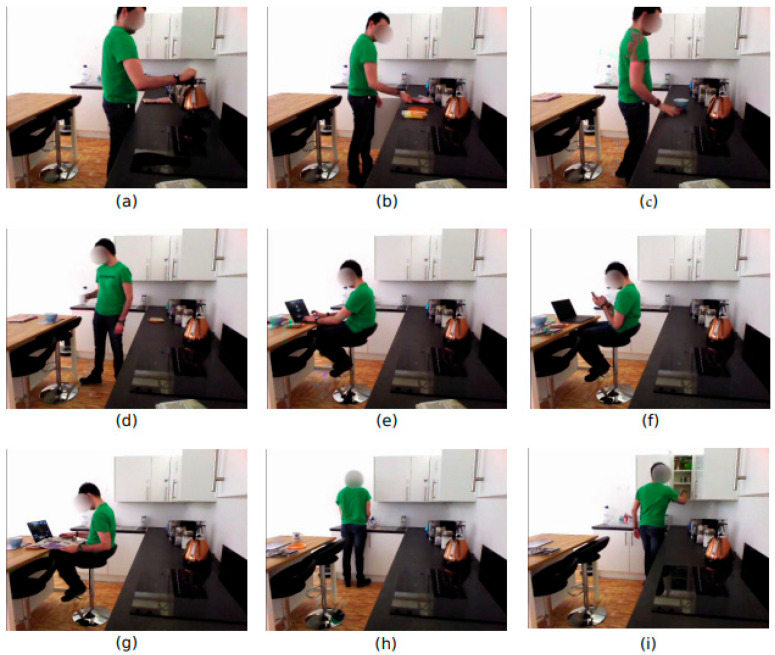
Sample frame sequences from the HWU-USP dataset.

A synthetic living-lab-based environment was used to capture the daily living activities of sixteen participants. In total, nine different ADLs have been performed in an assisted living facility inside Heriot-Watt University. The ADL performed include making a sandwich, making a bowl of cereal, making a cup of tea, setting the table, using a phone, reading a newspaper, using a laptop, cleaning the dishes, and tidying the kitchen. The data for each activity was recorded using three types of sensors, including IMU devices, ambient sensors, and videos. The ambient sensors, including four binary switches and PIR sensors, were placed in different places in the kitchen, such as switches, doors, mugs, dishes, and drawers. Inertial sensors, including MetaMotionR by MbientLab, were placed over the subjects’ dominant hands, wrist, and waist. For videos, a RGBD camera placed on the head of the TIAGo robot at VGA 640 × 480 and 25 fps has been used [33]. Figure 10 displays a few sample frame sequences from the selected dataset where a subject is (a) making a cup of tea; (b) preparing a sandwich; (c) preparing a bowl with cereals; (d) setting up the table; (e) using a laptop; (f) manipulating the cell phone; (g) reading a newspaper; (h) washing dishes; and (i) cleaning the kitchen. The dataset consists of multiple videos and inertial signals obtained from a waist clip and wristband.

#### 4.1.2. Opportunity++ Dataset

**Figure 11:**
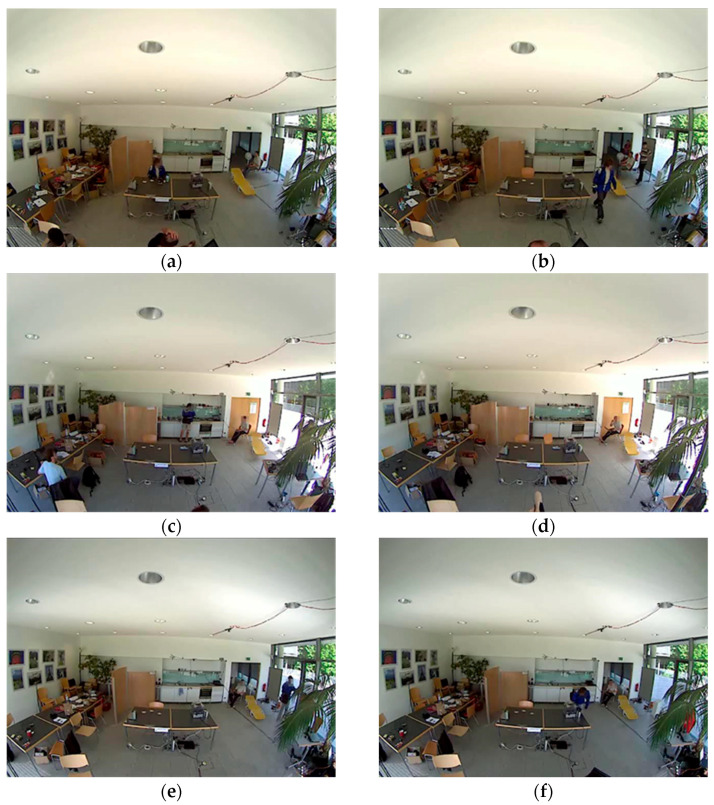
Sample frame sequences from Opportunity++.

This novel dataset is an addition to the previously proposed Opportunity [14] dataset. A total of 12 participants performed two types of activity drills. Two kinds of activities have been recognized through this dataset, such as locomotion-level and low-level activities. This system focused on 17 activities performed in different drills, including close door, open fridge, open door, close fridge, open dishwasher, open drawer, drink from cup, close drawer, close dishwasher, clean table, and toggle switch. The dataset was recorded using seven body worn IMU sensors, 12 object sensors, 13 ambient sensors, and videos. The body-worn sensors, including InertiaCube3 and Xsens MT9, were placed over the subject’s shoulders, wrists, elbows, waist, knees, and ankles. Whereas the ambient sensors, including 13 switches and 8 3D acceleration sensors, were attached to objects like milk, spoons, water bottles, glasses, drawers, and doors. Videos were recorded at 640 × 480 pixels and 10fps [34]. Figure 11 presents a few sample images captured during video recordings where a subject is (a) cleaning the table; (b) opening the door; (c) opening the drawer; (d) closing the door; (e) opening the door; and (f) closing the dishwasher.

### 4.2. Experimental Results

Locomotion prediction accuracy and skeleton point confidence levels for multimodal systems have been evaluated through the experiments. The proposed system attained sufficient outcomes due to robust multimodal IoT-based locomotion classification that shows significant improvement in the classification of ADL in terms of accuracy.

#### 4.2.1. Experiment 1: Via HWU-USP Dataset

The suggested methodology has been applied to the HWU-USP dataset. Table 1 displays the main ADL of the HWU-USP dataset, yielding a notable improvement in classification accuracy. The mean accuracy achieved for this experiment was 87.67%. Table 2 provides the confidence levels [35] calculated for each skeleton point extracted from the human silhouette. The distance is measured from the ground truth [36] for the HWU-USP and Opportunity++ datasets as:

(6) $$Dj=\sqrt{\sum_{n=1}^{M}{\left(\frac{X_{n}}{S_{n}}-\frac{Y_{n}}{S_{n}}\right)}^{2}}.$$

where $X_{n}$ gives the skeleton point position and $Y_{n}$ denotes the ground truth for both datasets. We chose a threshold of 15 to determine the recognition accuracy.

#### 4.2.2. Experiment 2: The Opportunity++ Dataset

The Opportunity++ dataset is utilized to test the experiments over the proposed multimodal IoT-based locomotion prediction system and is displayed using Table 3 in the form of a confusion matrix [37]. It is evident from the table that the offered methodology was able to attain an accuracy rate of 86.71% in this experiment. Table 4 explains the confidence levels and recognition accuracy measured against each skeleton point for the human silhouette extracted in our proposed methodology.

#### 4.2.3. Experiment 3: Evaluation Using Other Conventional Systems

Using the HWU-USP and Opportunity++ datasets, we have compared the proposed IoT-based multimodal locomotion prediction system with other similar multimodal systems present in the literature. The comparison of our approach with conventional multimodal state-of-the-art techniques is given in Table 5 [38]. An enhanced mean accuracy rate of 87.0% has been achieved through the proposed system when compared to the other conventional models.

## 5. Conclusions

A novel multimodal IoT-based state-of-the-art locomotion prediction system has been proposed in this article to reduce the errors caused by motion-related complexities and achieve an acceptable accuracy rate. Ambient, motion, and vision-based data have been retrieved from two publicly available datasets. Raw ambient data is pre-processed using a Butterworth filter; motion data is filtered through a Quaternion-based filter [44], and vision data is used to subtract background and extract the human silhouette. Next, we segmented the ambient and motion data along with extracting the skeleton points from the human silhouette for feature engineering over vision data. Then, we extracted Pearson correlation, LPCC, and SLIF features from ambient, motion, and vision data, respectively. Furthermore, the huge feature vector has been optimized using cross entropy and classified the data via RvNN. The system has been validated using confusion matrices, confidence levels, and skeleton point accuracies. The proposed multimodal IoT-based locomotion prediction model was able to achieve a mean accuracy rate of 87.0%. The results from these experiments have shown that our proposed system has outperformed several conventional multimodal approaches in the literature.

The proposed system did not perform well for complex motion patterns, such as tidying the kitchen or reading a newspaper. Therefore, we intend to further utilize more feature extraction methodologies and apply pattern recognition over motion and ambient sensor data to identify the static and dynamic motion signals before the feature extraction stage. It will be helpful in improving the proposed system for real-time applications.

## Figures and Tables

**Table 1:** Confusion matrix for locomotion classification for the proposed approach and the HWU-USP dataset.

	ms	tk	mbc	mct	st	up	rn	ul	cd
**ms**	**0.85**	0	0	0	0.05	0	0.1	0	0
**tk**	0	**0.86**	0	0.1	0	0	0	0	0.04
**mbc**	0.01	0	**0.89**	0	0	0.05	0	0.05	0
**mct**	0	0.1	0	**0.90**	0	0	0	0	0
**st**	0	0	0.12	0	**0.88**	0	0	0	0
**up**	0.04	0	0	0.07	0	**0.89**	0	0	0
**rn**	0	0	0	0	0.14	0	**0.86**	0	0.1
**ul**	0	0.03	0	0	0.1	0	0	**0.87**	0
**cd**	0	0	0.1	0	0	0.01	0	0	**0.89**

ms = making a sandwich; tk = tidying the kitchen; mbc = making a bowl of cereals; mct = making a cup of tea; st = setting the table; up = using a phone; rn = reading a newspaper; ul = using a laptop; cd = cleaning the dishes.

**Table 2:** Human skeleton points confidence level using the HWU-USP dataset.

Human Skeleton Points	Confidence Level	Distance	Recognition Accuracy
Head	0.81	13.6	0.91
Left shoulder	0.80	12.5	0.83
Right shoulder	0.77	11.2	0.75
Left elbow	0.69	14.5	0.97
Right elbow	0.74	13.6	0.91
Left wrist	0.80	9.7	0.65
Right wrist	0.78	10.8	0.72
Torso	0.80	13.1	0.87
Left knee	0.72	12.9	0.86
Right knee	0.75	11.7	0.78
Left ankle	0.66	12.4	0.83
Right ankle	0.68	11.9	0.79
**Mean Accuracy**	**0.75**		**0.82**

**Table 3:** Confusion matrix for locomotion classification for the proposed approach and the Opportunity++ dataset.

	od2	cd1	od1	cd2	cf	odw	of	cdw	cdr1	odr2	odr1	cdr2	odr3	ct	dc	cdr3	ts
**od2**	**0.89**	0	0	0.01	0	0.05	0	0	0.05	0	0	0	0	0	0	0	0
**cd1**	0	**0.85**	0.01	0	0	0	0.1	0	0	0	0	0	0	0	0.04	0	0
**cd1**	0.02	0	**0.87**	0	0	0	0	0.01	0	0.05	0	0	0.05	0	0	0	0
**cd2**	0	0.1	0	**0.87**	0	0	0	0	0.01	0	0.02	0	0	0	0	0	0
**cf**	0	0	0	0.05	**0.85**	0	0	0	0	0.1	0	0	0	0	0	0	0
**odw**	0	0	0	0	0.03	**0.90**	0	0	0	0	0	0	0	0	0.03	0	0.04
**of**	0	0	0	0	0	0	**0.84**	0	0	0	0	0.06	0	0	0	0.1	0
**cdw**	0	0	0	0	0	0.01	0	**0.85**	0.04	0	0	0	0.1	0	0	0	0
**cdr1**	0	0	0.12	0	0	0	0	0	**0.88**	0	0	0	0	0	0	0	0
**odr2**	0	0	0	0	0	0	0	0.06	0	**0.89**	0	0	0	0.05	0	0	0
**odr1**	0.1	0	0	0.1	0	0	0	0	0	0	**0.80**	0	0	0	0	0	0
**cdr2**	0	0.02	0	0	0.01	0	0	0	0	0	0	**0.87**	0	0	0	0	0.1
**odr3**	0	0	0	0	0	0.03	0	0	0.01	0	0	0	**0.86**	0	0.1	0	0
**ct**	0	0	0	0	0	0	0.1	0	0	0	0	0.04	0	**0.86**	0	0	0
**dc**	0	0	0	0	0	0	0	0	0	0	0.12	0	0	0	**0.88**	0	0
**cdr3**	0	0	0.02	0	0	0	0	0.03	0	0	0	0.05	0	0	0	**0.90**	0
**ts**	0	0	0	0	0	0	0.02	0	0	0.1	0	0	0	0	0	0	**0.88**

od2 = open door 2; cd1 = close door 1; od1 = open door 1; cd2 = close door 2; cf = close fridge; odw = open dishwasher; of = open fridge; cdw = close dishwasher; cdr1 = close drawer 1; odr2 = open drawer 2; odr1 = open drawer 1; cdr2 = close drawer 2; odr3 = open drawer 3; ct = clean table; dc = drink from cup; cdr3 = close drawer 3; ts = toggle switch.

**Table 4:** Human skeleton points confidence level using the Opportunity++ dataset.

Human Skeleton Points	Confidence Level	Distance	Recognition Accuracy
Head	0.85	14.2	0.95
Left shoulder	0.86	13.7	0.91
Right shoulder	0.85	12.9	0.86
Left elbow	0.79	11.5	0.77
Right elbow	0.78	13.2	0.88
Left wrist	0.74	10.9	0.73
Right wrist	0.69	12.7	0.85
Torso	0.87	11.2	0.75
Left knee	0.77	10.1	0.67
Right knee	0.79	14.0	0.93
Left ankle	0.60	11.1	0.74
Right ankle	0.59	12.6	0.84
**Mean Accuracy**	**0.76**		**0.83**

**Table 5:** Comparison of the proposed method with conventional systems.

IoT-based Multimodal Conventional Systems	Modalities	Accuracy
Memmesheimer et al. [39]	Ambient + Motion + Vision	0.86
Martínez-Villaseñor et al. [40]	Ambient + Vision	0.65
Piechocki et al. [41]	Ambient + Vision	0.74
Al-Amin et al. [42]	Motion + Vision	0.85
Gao et al. [43]	Ambient + Motion	0.83
**Proposed Multimodal IoT-based Locomotion Prediction System**	**Ambient + Motion + Vision**	**0.87**

## Data Availability

The data presented in this study are openly available in the Dryad Digital Repository, at https://doi.org/10.5061/dryad.v6wwpzgsj.
